# Supplementary material for: Integrative Methylome and Transcriptome Analysis Reveals Epigenetic Regulation of Pigmentation in Oujiang Color Common Carp
Source: Int J Mol Sci. 2025 Oct 14;26(20):10001. doi: 10.3390/ijms262010001 (PMC12562534; doi:10.3390/ijms262010001)
Supplement: Supplementary file 1 [file ijms-26-10001-s001.zip › Supplementary Materials.pdf]

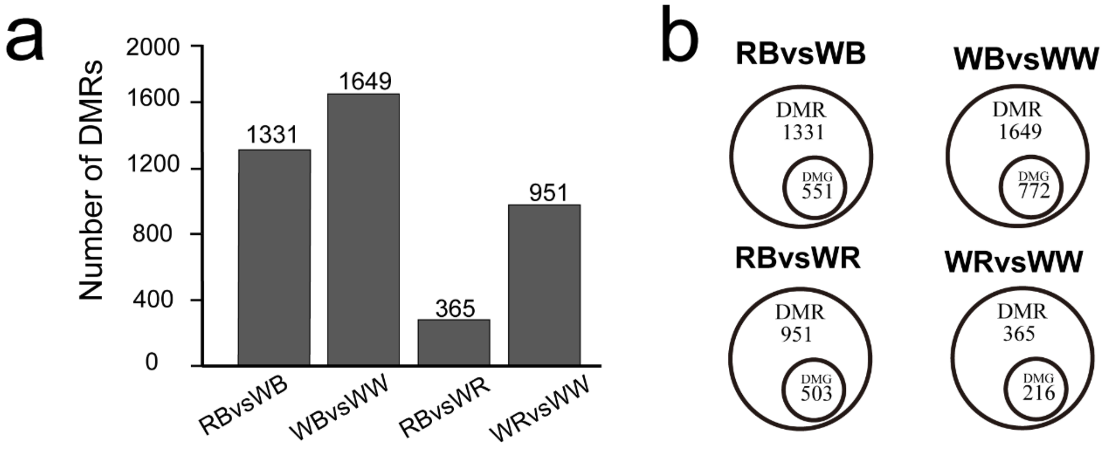


**Supplementary Figure S1. The number of DMRs and DMR-associated genes in each comparison group.** (a)The number of DMRs in each comparison group. (b) The number of DMR-associated genes in each comparison group.

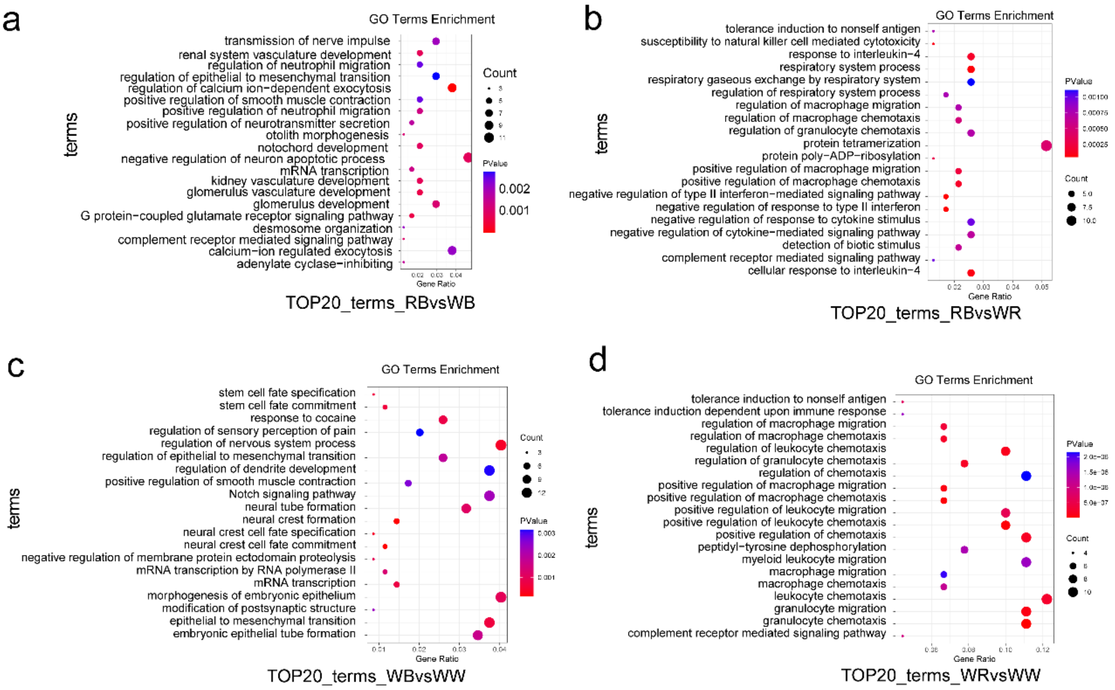


**Supplementary Figure S2. The TOP20 GO enrichment terms of DMGs in each group.** (a) The TOP20 GO enrichment terms of DMGs in RBvsWB. (b) The TOP20 GO enrichment terms of DMGs in RBvsWR. (c) The TOP20 GO enrichment terms of DMGs in WBvsWW. (d) The TOP20 GO enrichment terms of DMGs in WRvsWW.

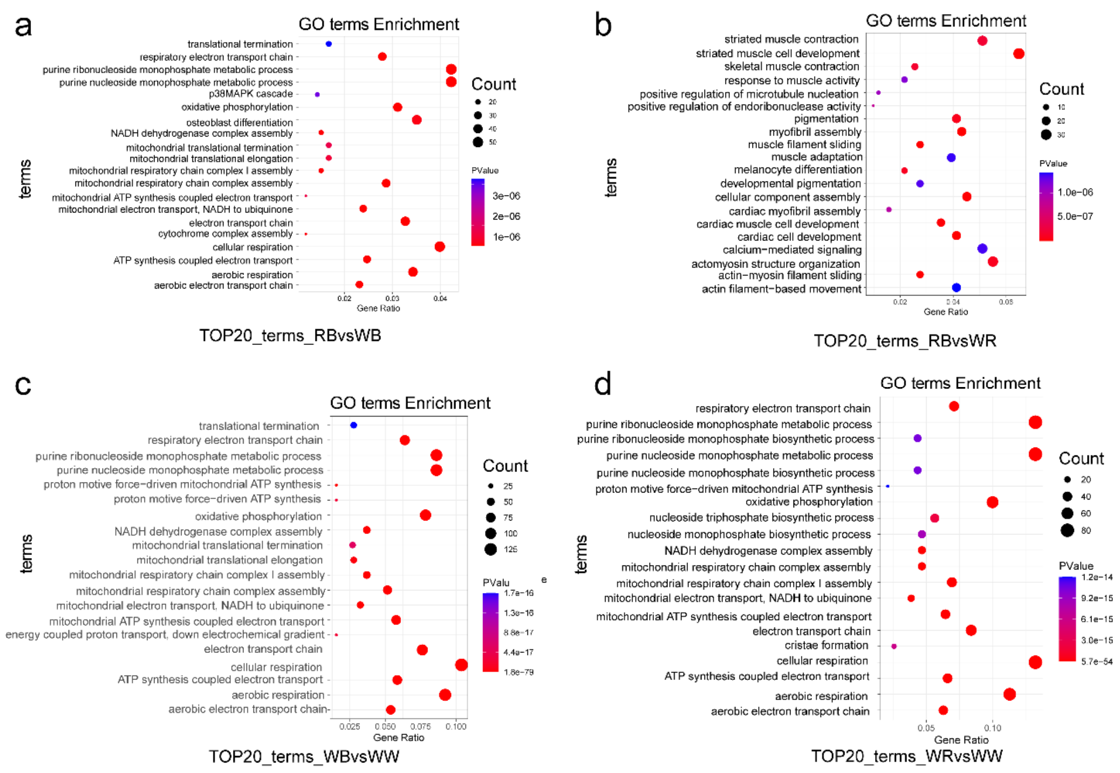


**Supplementary Figure S3. The TOP20 GO enrichment terms of DEGs in each group.** (a) The TOP20 GO enrichment terms of DEGs in RBvsWB. (b) The TOP20 GO enrichment terms of DEGs in RBvsWR. (c) The TOP20 GO enrichment terms of DMGs in WBvsWW. (d) The TOP20 GO enrichment terms of DEGs in WRvsWW.

**Supplementary Table S1. MBD-seq_quality_control_metrics**

| SampleID | Raw Reads | Clean Reads | Q30(%) |
|---|---|---|---|
| RB1 | 28,498,814 | 27,148,628 | 89.76% |
| RB2 | 40,437,721 | 38,931,104 | 90.12% |
| RB3 | 55,116,855 | 53,074,000 | 90.15% |
| WB1 | 31,330,174 | 30,243,676 | 90.07% |
| WB2 | 36,951,445 | 35,724,510 | 90.38% |
| WB3 | 32,517,977 | 31,236,791 | 89.72% |
| WW1 | 15,557,937 | 15,557,937 | 93.65% |
| WW2 | 42,500,345 | 40,752,767 | 91.66% |
| WW3 | 40,842,013 | 39,391,525 | 91.59% |
| WR1 | 26,968,065 | 26,377,136 | 92.08% |
| WR2 | 23,948,518 | 23,194,124 | 91.94% |
| WR3 | 21,006,809 | 20,234,406 | 91.76% |

**Supplementary Table S2. RNA-seq_quality_control_metrics**

| SampleID | Raw Reads | Clean Reads | Q30(%) |
|---|---|---|---|
| RB1 | 25,686,388 | 24,609,068 | 91.45% |
| RB2 | 28,241,420 | 27,682,050 | 90.85% |
| RB3 | 22,805,740 | 22,159,402 | 90.21% |
| WB1 | 24,766,382 | 23,783,860 | 91.26% |
| WB2 | 26,337,432 | 25,824,716 | 90.98% |
| WB3 | 21,428,528 | 20,971,270 | 90.81% |
| WW1 | 39,642,101 | 38,764,648 | 88.35% |
| WW2 | 25,190,430 | 24,652,398 | 90.80% |
| WW3 | 34,148,372 | 33,590,668 | 91.12% |
| WR1 | 20,178,300 | 19,862,756 | 91.11% |
| WR2 | 21,912,906 | 21,173,668 | 90.95% |
| WR3 | 32,459,772 | 31,629,760 | 91.26% |
